# Upfront Screening by Quantitative Real-Time PCR Assay Identifies NUP98::NSD1 Fusion Transcript in Indian AML Patients

**DOI:** 10.3390/diagnostics12123001

**Published:** 2022-12-01

**Authors:** Arunim Shah, Akhilesh Sharma, Shobhita Katiyar, Anshul Gupta, Chandra Prakash Chaturvedi

**Affiliations:** 1 Stem Cell Research Center, Department of Hematology, Sanjay Gandhi Postgraduate Institute of Medical Sciences, Raebareli Road, Lucknow 226014, India; 2 Department of Hematology, Sanjay Gandhi Postgraduate Institute of Medical Sciences, Raebareli Road, Lucknow 226014, India

**Keywords:** *NSD1*, AML, NUP98, *HOX* Cluster, *FLT3*, *FLT3-ITD*

## Abstract

*NUP98::NSD1* fusion, a cryptic translocation of t(5;11)(q35;p15.5), occurs predominantly in pediatric AML, having a poor prognostic outcome. There are limited studies on the diagnosis of *NUP98::NSD1* fusion in a clinical setting, and most of the data are from Western countries. No study on the detection of this translocation has been reported from the Indian subcontinent to date. One possible reason could be the lack of availability of a potential tool to detect the fusion transcript. We have developed a real-time quantitative PCR (qRT-PCR)-based assay to detect *NUP98::NSD1* fusion transcript with high sensitivity and specificity. Screening 150 AML patients (38 pediatric and 112 adults) using the assay showed the presence of fusion transcript in six patients including 03 pediatric, and 03 adult patients. We observed a prevalence rate of 7.89% (3/38) and 2.67% (3/112) fusion transcript in pediatric and adult patients, respectively. Sanger sequencing further validated the occurrence of *NUP98::NSD1* fusion in all six patients. Molecular characterization of these patients revealed a co-occurrence of *FLT3-ITD* mutation, accompanied by altered expression of the *HOX* and other genes associated with AML. All six patients responded poorly to induction therapy. Overall, this is the first study to show the presence of the *NUP98::NSD1* fusion transcript in Indian AML patients. Further, we demonstrate that our in-house developed qRT-PCR assay can be used to screen *NUP98::NSD1* fusion in clinical settings.

## 1. Introduction

*NSD1* (nuclear receptor binding SET domain-containing protein 1) is a histone methyltransferase that mediates gene activation [1]. *NSD1* is vital for normal growth and development, and any alteration in the expression of NSD1 can lead to a developmental defect such as SOTOS syndrome [2] and cancers, including neuroblastoma, glioblastoma [3], head and neck squamous cell carcinoma (HNSCC) [4], lung carcinoma [5], renal carcinoma [6], and hematological cancers such as acute myeloid leukemia (AML) (6). In AML, *NSD1* on chromosome 11 undergoes a chromosomal translocation with *Nucleoporin 98 (NUP98)* on chromosome 5 to form a fusion transcript *NUP98::NSD1* [t(5;11)(q35;p15.5)] [7]. The oncogenic fusion transcript is generated by the fusion of amino-terminal FG repeat domains of *NUP98* to the carboxyl-terminal region of *NSD1* that contains a 5-PHD finger repeat, a PHD finger-like Cys-His rich domain, a PWWP domain, and a HMT domain [8,9]. The breakpoint in the fusion gene consistently corresponds to exon 12 of *NUP98* (1407 nucleotide from transcription start site) and exon 6 of *NSD1* (3935 positions from ATG start codon up to the end (stop codon) of the gene) [9]. The fusion protein has an overall prevalence of 4–5% in pediatric AML and 1.2–3% in adult AML, with a poor prognostic outcome [9,10,11,12]. *NUP98::NSD1* translocation is found to co-occur with other types of mutation, namely, *FLT3* internal tandem duplication (*FLT3*-ITD), Wilms’ tumor suppressor gene1 (WT1), neuroblastoma RAS *(NRAS), CCAAT enhancer binding protein alpha (CEBPA)* and *MYC*, of which the most frequent co-occurrence is observed with *FLT3-*ITD, which accounts for greater than 80% of the total *NUP98::NSD1* positive cases [10]. The patients showing co-occurrence of *FLT3*-ITD mutation with *NUP98::NSD1* have a poorer outcome than those expressing either *NUP98::NSD1* or *FLT3*-ITD mutations. It was observed that four-year event-free survival was less than 10% in pediatric and adult cases with NUP98-NSD1 fusion, which turned even worse when co-occurring with *FLT3*-ITD [10]. Though there are few studies from Western countries [11,13,14] and other parts of the world [15] on the prediction and prevalence of *NUP98::NSD1* in AML patients, nevertheless, the prevalence of this fusion protein remains unknown in Indian AML patients, possibly due to the non-availability of reliable screening methods.

Therefore, the present study was designed with the following objectives: (i) to develop a RT-qPCR-based assay to detect and determine the copy number of *NUP98::NSD1* [t(5;11)(q35;p15.5)] transcript with high sensitivity and specificity; (ii) to use this assay for routine screening to find out the presence of this fusion transcript in AML patients.

## 2. Materials and Methods

### 2.1. Sample Collection

Peripheral blood (3 mL) was collected after taking written informed consent from adult AML patients, legally accepted guardians of pediatric AML patients, and five healthy donors. The study was approved by the Institutional Ethics Committee (IEC code: 2021-12-SRF-118). The clinical information of the patients was collected from the hospital information system (HIS) of the Institute.

### 2.2. Plasmid Construction

*NUP98* breakpoint region (1408 bp) and *NSD1* breakpoint region (4295 bp) were PCR amplified using *NUP98::NSD1* forward and *NUP98::NSD1* reverse primers (Table 1) using GenScript^®^, Piscataway, NJ, USA *NUP98* (Clone ID OHu26540D) and *NSD1* (Clone ID OHu18754D) plasmids as PCR templates. The *NUP98::NSD1* fusion fragment was ligated in pcDNA5/TO vector using GenScript^®^, Piscataway, NJ, USA GenBuilder^TM^ cloning kit (Cat no. L00701-10), as per the manufacturer’s instructions. The cloning of the fusion gene in the pcDNA5/TO was confirmed by restriction–digestion analysis and sanger sequencing.

### 2.3. Designing of Primers and Probes

Primers and probes were designed using ABI Primer Express 3 software to detect the breakpoint fusion region of *NUP98::NSD1* and *Abelson tyrosine-protein kinase 1 (ABL1)* as an internal control. These primers and FAM/VIC- labelled probes (Table 1) were used for Taqman quantitative RT-PCR in a single multiplex reaction.

### 2.4. Sensitivity of Assay

The assay’s sensitivity was determined by qRT-PCR of serially diluted plasmids from 12.5 ng with 10 folds of serial dilution until no detectable relative fluorescence was observed in qRT PCR [16].

### 2.5. Specificity of the Assay

The specificity of the *NUP98::NSD1* qRT-PCR assay was determined by qRT-PCR, as previously described [17]. Briefly, a known positive sample for *NUP98::NSD1* fusion was serially diluted 10-fold from 100 ng to 0.01 ng. The total concentration of cDNA was maintained at 100 ng in each diluted fraction by healthy control cDNA.

### 2.6. RNA Extraction and cDNA Preparation

Total RNA was extracted using the Purelink^TM^ RNA extraction kit (Thermo Fisher Scientific, Waltham, MA, USA) as per the manufacturer’s protocol. Two micrograms of RNA were used to prepare cDNA using a high-capacity cDNA synthesis kit (Applied Biosystems^TM^, Waltham, MA, USA), as per the manufacturer’s instructions. The cDNA was used for Sanger sequencing, and qRT-PCR expression analysis of HOX genes and other genes involved in *NUP98::NSD1* mediated leukemogenesis.

### 2.7. Quantitative qRT-PCR

Real-time quantitative PCR was conducted on BioRad CFX 96 real-time instrument using Taq-Man probes (Applied Biosystems^TM^, Waltham, MA, USA) and gene-specific primers to detect *NUP98::NSD1* and *ABL1* transcripts, whereas minor groove binder EVA-green for HOX and other genes transcript estimation was also considered. The *ABL1* gene was used for normalization in the NUP98-NSD1 quantitation assay, while *GAPDH* was used as an internal control for the relative quantification of *HOX* genes and other gene transcripts. The relative quantification was conducted, as described previously [18]. The primers and probes used for qRT-PCR are mentioned in Table 1.

### 2.8. Statistical Data Analysis

All the data are expressed as the mean ± standard deviation (SD). Statistical significance was determined using a Student *t*-test. Three independent biological replicates were used for statistics, and standard deviation was calculated to generate error bars.

### 2.9. Sanger’s Sequencing for Validation of NUP98-NSD1 Fusion

*NUP98::NSD1* transcripts were PCR amplified using specific primers (Table 1). PCR product was purified using the QIAquick PCR purification kit (Qiagen, Hamburg, Germany). The cycle sequencing of purified PCR products was conducted using 1 pmol primer and Big Dye terminator cycle sequencing kit v3 (Applied Biosystems^TM^, Waltham, MA, USA). Cycle sequencing products were purified using the Big Dye X-terminator kit (Applied Biosystems^TM^, Waltham, MA, USA), and the purified products were run on 3500 Genetic Analyzer ABI 3500 (Applied Biosystems^TM^, Waltham, MA, USA) for capillary electrophoresis. The data obtained were analyzed for the presence of fusion transcript in patients’ samples by aligning the sequence data using the NCBI blast tool.

### 2.10. FLT3-ITD Fragment Analysis

Genomic DNA was extracted from peripheral blood using the QIAamp DNA mini kit (Qiagen, Hamburg, Germany). The catalytic domain of the *FLT3* gene was PCR amplified for fragment analysis to detect ITD mutation using a 5′ labelled FAM forward primer and a reverse primer (Table 1). Fragment analysis of PCR products was conducted by sizing-standard LIZ 600 using 3500 Genetic Analyzer ABI 3500 (Applied Biosystems^TM^, Waltham, MA, USA). The data obtained were analyzed using Gene Mapper v5.0 (Applied Biosystems^TM^, Waltham, MA, USA). The allelic ratio (AR) ratio was calculated, as previously described [19].

## 3. Results

### 3.1. Development of a qRT-PCR Assay to Determine NUP98::NSD1 Fusion Transcript

**Figure 1:**
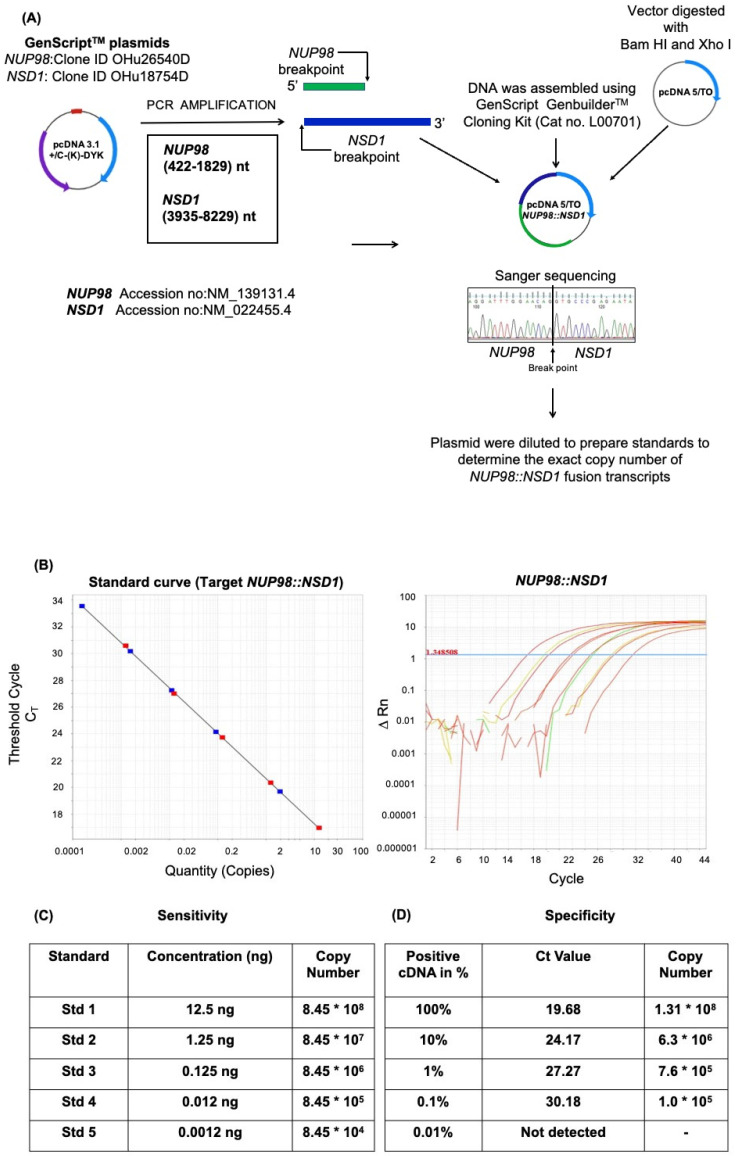
(**A**) Schematic representation of molecular cloning and validation strategy for *NUP98::NSD1* DNA sequence in expression vector pcDNA/TO5 plasmid vector. The plasmid with the *NUP98::NSD1* gene was used as a positive control/standard for developing the quantitative assay. (**B**) Development of the *NUP98::NSD1* screening assay. This figure shows the standard curve plot obtained with a slope of −3.389 with an R^2^ (correlation coefficient) of 1. The variation of the Ct between the replicates was found to be <0.5 Ct, and the standard deviation was <0.15. The table represents the known concentration of standards with their corresponding copy number detected using qRT-PCR. (**C**) The sensitivity of the assay was determined by serially diluting the *NUP98::NSD1* plasmid. (**D**) The assay’s specificity was determined by serially diluting the *NUP98::NSD1* positive patient sample with healthy donor cDNA, as described in the results.

The cloning of the fusion gene in the pcDNA/TO5 expression vector and the validation were conducted, as presented in Figure 1A. For copy number detection, the plasmid containing the *NUP98::NSD1* fusion gene was serially diluted to obtain five standards corresponding to the copy number of *NUP98::NSD1* fusion depicted using qRT-PCR (Figure 1B). The copy number was mathematically calculated by the calculator for determining the number of copies of a template, as per the formula [number of copies = (amount of DNA in ng × 6.022 × 10^23^)/(length of templet (bps) × 1 × 10^9^ × 650)] mentioned on the website http://cels.uri.edu/gsc/cndna.html (accessed on 11 August 2020) by URI Genomics & Sequencing Center. To determine the sensitivity of our qRT-PCR assay for the detection of *NUP98::NSD1* fusion, the standard curve was plotted against five standards of variable concentration ranging from 12.5 to 0.00125 ng (Figure 1C). We found that the lower limit of detection of standard control in our assay was 0.00125 ng, which corresponds to log10^−4^. As per the CLSI/NCCLS EP17-a guideline, the minimum required sensitivity of a qRT-PCR assay should be log10^−3^. Thus, our in-house developed assay had a sensitivity as per international standards.

To assess the specificity of our assay, a known positive cDNA sample for *NUP98::NSD1* fusion was serially diluted 10-fold from 100 ng to 0.01 ng with a healthy control cDNA, such that the final concentration of total cDNA was 100 ng in all diluted samples. 100% cDNA corresponds to 100 ng of *NUP98::NSD1* of the positive patient; likewise, 10% cDNA corresponds to 10 ng of *NUP98::NSD1* of the positive cDNA plus 90 ng of healthy individual cDNA. Similarly, the dilution series was established for other fractions. On the log scale, 100% cDNA corresponds to log 10 of the transcript, while 0.01% cDNA corresponds to log 10^−4^ of the transcript. Our qRT-PCR assay for *NUP98::NSD1* fusion can detect up to log 10^−3^ of the transcripts, corresponding to 0.1 ng of positive cDNA of the sample (Figure 1D). Thus, the specificity of the limit of detection of *NUP98::NSD1* fusion transcript in our assay is 0.1 ng or log10^−3^ of the transcript.

Further, the amplification efficiency of our assay was calculated using a serial dilution of our target cDNA samples followed by qRT-PCR. The Ct values were plotted on the logarithmic scale along with the corresponding concentrations, and a linear regression curve was plotted to calculate a slope line. The efficiency of the assay was calculated using the formula E = −1 + 10^(−1/slope)^ and was 97.25%.

### 3.2. Screening of Patients for the Presence of NUP98::NSD1 Fusion in the Indian Cohort

**Figure 2:**
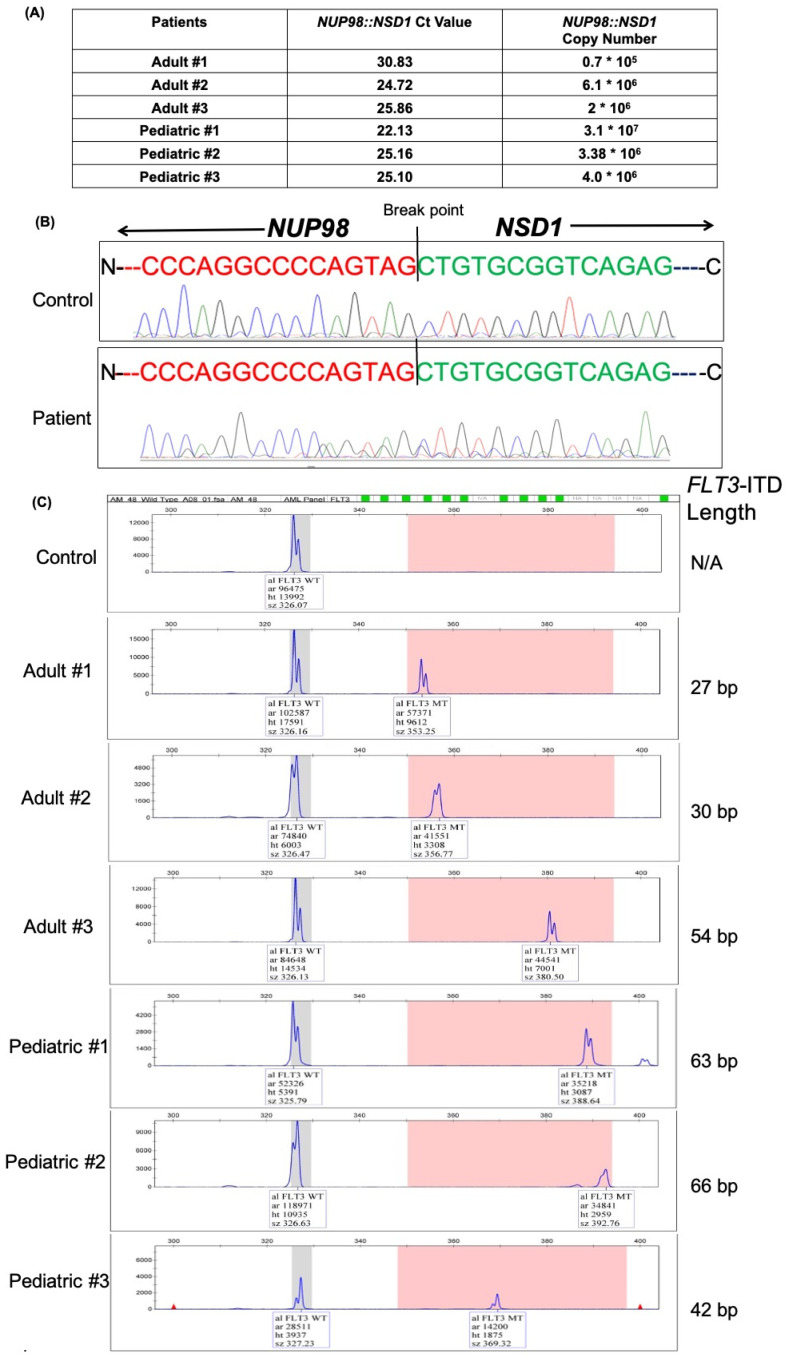
Screening and validation of AML patients (**A**) qRT PCR data revealed the presence of *NUP98::NSD1* fusion transcript. The table shows the Ct value and their corresponding *NUP98::NSD1* copy number. (**B**) Fluorescent peak trace of chromatograms obtained after Sanger sequencing showing the breakpoint of *NUP98::NSD1* fusion transcript. Exon 12 of *NUP98* was fused to exon 6 of *NSD1* in all six patients. The plasmid with *NUP98::NSD1* fusion was taken as a positive control. (**C**) Fragment analysis of *FLT3*-ITD mutation in *NUP98::NSD1*^+ve^ patients revealed all six patients had co-occurrence of *FLT3*-ITD mutation. The peak in the grey bin indicates wild-type *FLT3*, while the peak in the pink bin indicates mutant *FLT3*.

To demonstrate the utility of our in-house developed qRT-PCR assay to detect the presence of *NUP98::NSD1* oncogenic fusion in patients, we screened 150 acute myeloid leukemia (AML) patients. Among 150 AML patients, six AML patients showed the expression of *NUP98::NSD1* fusion transcript (Figure 2A). Three out of 112 adults and three out of 38 pediatric patients were positive for the fusion transcript, indicating a prevalence rate of the fusion transcript of 2.67% and 7.89% in adult and pediatric AML cases, respectively. All the positive patients had a blast count of more than 50%.

### 3.3. Sanger Sequencing Confirmation of NUP98::NSD1 Breakpoint Fusion

To further confirm and validate the *NUP98::NSD1* fusion transcript identified by qRT-PCR assay in patients, we sequenced the fusion region of *NUP98::NSD1* in all six positive patients using Sanger sequencing. All the patients had the common breakpoint of exon 12 of *NUP98* (1407 nucleotide from transcription start site) and exon 6 of *NSD1* (3935 positions from ATG start codon till 8229 position), as shown in Figure 2B. This breakpoint was comparable to our positive control and previously reported literature [13,14,20].

### 3.4. FLT3-ITD Status in NUP98::NSD1 Fusion Positive Patients

Internal tandem duplication of FLT3 has been reported to co-occur in 85–90% of *NUP98::NSD1*^+ive^ cases [19,21]. Therefore, fragment analysis of *FLT3*-ITD with Nup98-NSD1 in six positive patients was performed to determine the co-occurrence of *FLT3*. Fragment analysis revealed that all six patients with *NUP98::NSD1* fusion had co-occurrence of *FLT3*-ITD mutation. Adult patients 1 and 2 had 27 bp and 30 bp *FLT3*-ITD, respectively. Adult patient 3 had 54 bp *FLT3*-ITD. In the case of pediatric patients, pediatric patient 1 had 63 bp *FLT3*-ITD, pediatric patient 2 had 66 bp FLT3-ITD, and pediatric patient 3 had 42 bp FLT3-ITD (Figure 2C).

### 3.5. NUP98::NSD1 Fusion Patients Have Altered the Expression of Genes Associated with Self-Renewal and Differentiation

**Figure 3:**
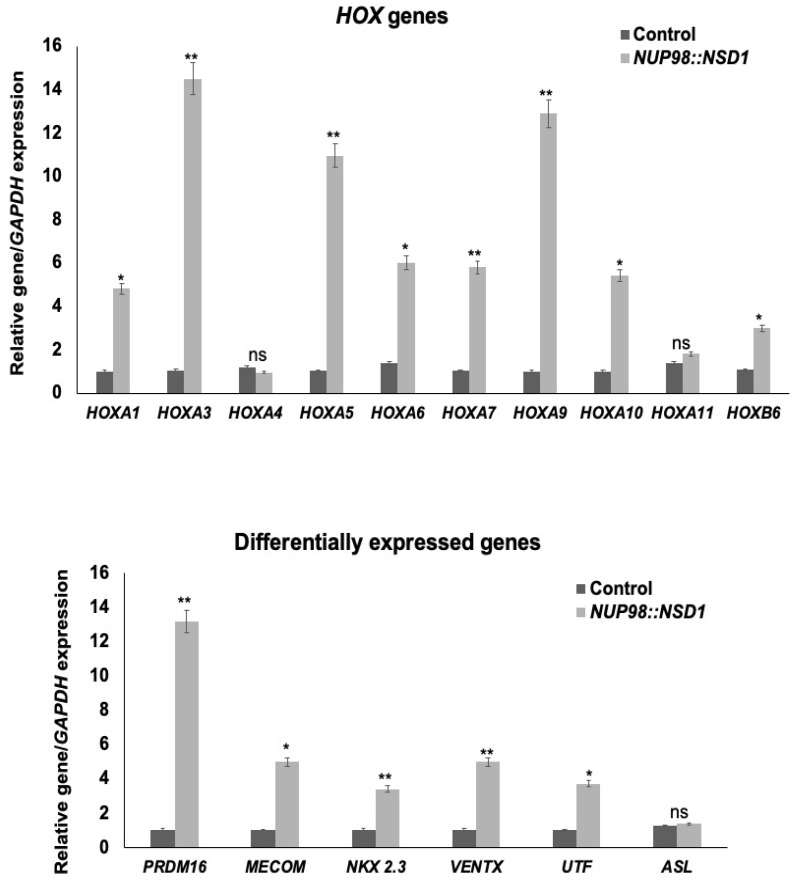
Patients with NUP98-NSD1 fusion had an altered gene expression profile. This figure shows an alteration in the expression of HOX cluster and other genes associated with self-renewal and differentiation in patients with NUP98-NSD1 oncogenic fusion. *HOX* genes (*HOXA*1*, A3, A4, A5, A6, A7, A9, A10, A11,* and *B6*) and other genes (*PRDM16, MeCOM, NKX 2.3, VENTX, ASL,* and *UTF)* involved in self-renewal were analyzed at the mRNA level by real-time RT-qPCR in healthy control vs. *NUP98::NSD1*^+ive^ patients. Transcripts are expressed relative to GAPDH. The average values from 06 *NUP98::NSD1* patients vs. six healthy controls in three replicate experiments are represented with error bars corresponding to SD. * *p* < 0.05; ** *p* < 0.01; ns: non-significant.

*NUP98::NSD1* translocation is associated with the deregulation of the *HOX* gene clusters [9,22]. To assess if our patients with *NUP98::NSD1* fusion also have altered expression of *HOX* genes, we studied the expression of *HOX* gene clusters in these patients. Of the eight of ten *HOX* genes tested—*HOXA1* (fold increase = 5), *HOXA3* (fold increase = 14), *HOXA5* (fold increase = 10), *HOXA6* (fold increase = 6), *HOXA7* (fold increase = 6), *HOXA9* (fold increase = 13), *HOXA10* (fold increase = 6), and *HOXB6* (fold increase = 3)—were upregulated (Figure 3). No significant change was observed for *HOXA4* and *HOX A11* genes. In addition to *HOX* genes, we also tested for gene expression profiles of the other representative genes of acute myeloid leukemia (AML). We found that *PRDM16* (fold increase = 13) and *MECOM* (fold increase = 5), an alternatively spliced variant of *MDS1/EVI1*, highly homologous to *PRDM16*, were highly upregulated. We also found a five-fold increase in the expression of the *VENTX* gene in *NUP98::NSD1*-positive patients, while *UTF* and *NKX2-3* were also upregulated more than three-fold in these patients (Figure 3). The fold increase in upregulated genes was plotted as average mean values of all patients with standard deviation.

### 3.6. NUP98::NSD1 Patients Show Poor Responses to Induction Therapy

The *NUP98::NSD1* patients are reported to show poor outcomes to induction therapy and/or hematopoietic stem cell transplantation (HSCT) [10,23]. In the current study, all six NUP98-NSD1 patients had a high WBC count (Average mean WBC count 18.06 × 10^9^/L), with % of blast cells greater than 50% at diagnosis. All six patients have *FLT3*-ITD co-occurrence with an AR (allelic ratio) less than 0.4 (Table 2) and were treated as per the standard treatment guidelines [24]. Five patients were treated with 3 + 7 (daunorubicin + cytosine arabinoside (ara-C)) combination induction chemotherapy, while one patient received azacitidine + venetoclax in view of poor performance status at presentation. Of the six *NUP98::NSD1* positive patients, only one patient achieved complete remission to induction chemotherapy, while five patients failed induction chemotherapy and had to be administered salvage chemotherapy. All five patients who failed induction chemotherapy succumbed to sepsis due to severe febrile neutropenia. Further, one patient who responded to induction chemotherapy underwent an allogenic stem cell transplant but relapsed seven months post-transplant (Table 2).

## 4. Discussion

AML patients with *NUP98::NSD1* fusion have a poor prognosis, high induction failure, and poor survival [10,19]. Since the fusion is cytogenetically cryptic, it is not detected in the karyogram during conventional karyotyping [25]. Therefore, a robust and reliable method is required to screen and identify these patients during diagnosis. Fewer studies have used qRT-PCR-based techniques using SYBR Green/TaqMan assay to screen this fusion [26]; however, the key limitation with these assays was the lack of a quantitative method to determine the copy number of the fusion transcript. We have developed a reliable qRT-PCR based multiplex assay with high sensitivity and specificity for detecting *NUP98::NSD1* transcripts in patient samples. The assay has a log 10^−3^ sensitivity corresponding to the detection of 0.1 ng of the fusion transcript. Furthermore, the assay is highly specific to detect only the fusion transcript, as no *NUP98::NSD1* fusion transcript was detected up to 100 ng of negative cDNA sample. The assay’s limit of quantification (LOQ) and limit of detection (LOD) was at par with the commercially available kits, as previously described [16,17]. Thus the assay can be used for the routine investigation to see the presence of the *NUP98::NSD1* fusion transcript for newly admitted patients, specifically AML patients showing the *FLT3*-ITD and not responding to induction therapy. Additionally, it can be used as a tool for MRD detection in the future.

After successfully developing the qRT-PCR assay for *NUP98::NSD1* fusion detection, our goal was to use it to detect fusion transcripts in the Indian cohort of AML patients. The screening of 150 AML patients using the assay identified six patients with *NUP98::NSD1* translocation, which included three adults and three pediatric patients. While the worldwide data shows a 4–5% prevalence rate of *NUP98::NSD1* in pediatric AML and 1.3–3% in adult AML [9,10,11,12], we found that the percentage prevalence of *NUP98::NSD1* fusion transcript in our pediatric was 7.89% (3/38), which was comparatively higher than reported previously. However, this difference could be because of the smaller sample size in our case. Studies on a larger cohort of pediatric patients will be helpful in the evaluation of NUP98::NSD1 prevalence in pediatric patients. The rate of *NUP98::NSD1* positivity in adult patients was 2.67% (3/112), which concords with the published studies [10,11,12]. However, this is the first study from the Indian subcontinent to demonstrate the presence and prevalence of *NUP98::NSD1* transcripts in AML patients.

*NUP98::NSD1* translocation is reported to associate with the co-occurrence of *FLT3*-ITD, with more than 85% of *NUP98::NSD1* patients having *FLT3*-ITD [10,19]. To see if our *NUP98::NSD1* patients also have the co-occurrence of *FLT3*-ITD, we studied the presence of this rearrangement using fragment analysis. We found that all 06 *NUP98::NSD1* positive patients have co-occurrence of *FLT3*-ITD, which was in line with published studies [12,19,27].

To develop effective therapeutic strategies for *NUP98::NSD1* leukemogenesis, a clear understanding of the mechanistic basis of *NUP98::NSD1* leukemogenesis needs to be well characterized. The gene expression profile of *NUP98::NSD1* patients has revealed an alteration in the genes associated with *HOX* gene clusters [9,22]. We also found that *HOX* genes were highly upregulated in all the *NUP98::NSD1* patients, specifically *HOX A1, HOXA3, HOXA5, HOXA6, HOX A7*, *HOX A9*, *HOX A10,* and *HOXB6* expression, suggesting that the alteration is conserved in our AML patients with *NUP98::NSD1*. The increased expression of *HOX* genes has been reported to promote stem cell self-renewal and to block terminal differentiation, thus leading to *NUP98::NSD1*-mediated leukemogenesis [9].

We also found that several other genes associated with AML were significantly altered in *NUP98::NSD1* patients. The high-level expression of *PRMD16* is a key clinical feature of AML [28,29]. We found that *PRDM16*, along with homologous protein *MECOM*, is upregulated in *NUP98::NSD1* patients. Functionally, *PRDM16* is essential for hematopoietic stem cell maintenance, and deregulation of *PRDM16* induces leukemogenesis [28]. Several reports conclude that high *PRDM16* expression is a crucial marker for poor prognosis in AML patients [28]. Similarly, *VENTX*, an oncogenic transcription factor that promotes myeloid differentiation, was also upregulated in *NUP98::NSD1* patients, suggesting its role in leukemogenesis [30]. The *UTF* gene that is expressed during embryonic development, along with NKX2-3, were also upregulated in *NUP98* AML patients [30]. These results suggest that our AML patients with *NUP98::NSD1* transcript showed altered genomic profile, deregulated expression of *HOX* cluster, and other leukemic genes, as previously described [9,22].

It has been reported that *NUP98::NSD1* is an independent predictor of poor prognosis [9]; however, frequent co-occurrence of *FLT3*-ITD and *NUP98::NSD1* have a poorer prognosis, thus raising the concern about whether this poor prognostic outcome is because of *NUP98::NSD1* or determined by the co-occurrence of *FLT3*-ITD. The AR (allelic ratio) of *FLT3*-ITD also has a prognostic significance in AML [19]. Patients with FLT-ITD AR greater than 0.4 are categorized as high-risk patients with high chances of induction failure [19]. In the current study, all six patients were detected with *FLT3*-ITD AR of less than 0.4; still, these patients showed poor responses to the induction therapy and/or to HSCT. These results suggest that the co-occurrence of *NUP98::NSD1* fusion in low-risk *FLT3*-ITD patients with AR less than 0.4 may transform these patients into a high-risk category showing poor clinical outcome and induction therapy failure, thus speculating that the presence of NUP98-NSD1 may cause disease severity, poor drug response, and a high rate of induction failure. Therefore, these patients should be robustly screened and categorized as a new subset of AML patients. Furthermore, more in-depth molecular characterization of these patients is necessary to understand the disease’s pathobiology and develop novel treatment protocols. However, our findings should be validated in larger prospective cohort studies. Overall, the current study, for the first time, reports the identification and prevalence of *NUP98::NSD1* fusion transcript in Indian AML patients.

## 5. Conclusions

In summary, we have developed a qRT-PCR assay with high sensitivity and specificity to determine the copy number of the *NUP98::NSD1* fusion transcript. The assay can robustly screen the *NUP98::NSD1*-positive AML patients with absolute copy number detection. Furthermore, our data suggest that the *NUP98::NSD1* fusion co-occurs with *FLT3*-ITD and worsens the prognosis of *FLT3-*ITD positive patients independent of *FLT3*-ITD allelic ratio; hence it should be routinely screened in all *FLT3*-ITD-positive AML patients, as it has important treatment ramifications.

## Figures and Tables

**Table 1:** List the primers and probes used for cloning, sequencing, expression analysis, and fragment analysis.

Cloning Primers	
*NUP98::NSD1* fusion forward	5′AAAGATCATGACATAGATTACAAGGATGACGATGA CAAGGCCATGTTTAACAAATCATTTGGAACACC-3′
*NUP98::NSD1* fusion reverse	5′AGTCGAGGCTGATCAGCGGGTTTAAACGGGCC CTCTAGACCTACTTCTGTTCTGATTCTGCACACTT-3′
**Sequencing Primers**	
*NUP98-*Seq F	5′-ACTCTTGGAACTGGGCTTGG-3′
*NSD1*-Seq R	5′-GGCTAGAAGGCTTTCCTCTTC-3′
**qRT-PCR primers**	
*NUP98* t(5,11) F	5′-GGCCCCTGGATTTAATACTACGA-3′
*NSD1* t(5,11)R	5′-CTTCCTAAGGCGTTTCTTCTCTGA-3′
*NUP98-NSD1* t(5,11) probe	5′-FAM-TTTGGAGCCCCCCAGGCC-MGB NFQ-3′
*ABL1*-F	5′-CCCAGAGAAGGTCTATGAACTCATG-3′
*ABL1*-R	5′-AGGAGGGCCGGTCAGA-3′
*ABL1* probe	5′-VIC-TCCACTGCCAACATGC-MGB NFQ-3′
*HOXA1* F	5′-CCCTCGGACCATAGGATTACAA-3′
*HOXA1* R	5′-GCCGCCGCAACTGTTG-3′
*HOXA3* F	5′-CGACAGCTCGGCGATCTAC-3′
*HOXA3* R	5′-CGGGTACGGCTGCTGATT-3′
*HOXA4* F	5′-GGTGGTGTACCCCTGGATGA-3′
*HOXA4* R	5′-GACTTGCTGCCGGGTATAGG-3′
*HOXA5* F	5′-GGAGTTCCACTTCAACCGTTACC-3′
*HOXA5* R	5′-CGGAGAGGCAAAGAGCATGT-3′
*HOXA6* F	5′-GTCTGGTAGCGCGTGTAGGT-3′
*HOXA6* R	5′-CCCTGTTTACCCCTGGATG-3′
*HOXA7* F	5′-CTTCTCCAGTTCCAGCGTCT-3′
*HOXA7* R	5′-AAGCCAGTTTCCGCATCTAC-3′
*HOXA9* F	5′-CCACGCTTGACACTCACACT-3′
*HOXA9* R	5′-GCTCTCATTCTCGGCATTGT-3′
*HOXA10* F	5′-TCTTTGCTGTGAGCCAGTTG-3′
*HOXA10* R	5′-CTCCAGCCCCTTCAGAAAAC-3′
*HOXA11* F	5′-CGGCCACACTGAGGACAAG-3′
*HOXA11* R	5′-AACTCTCGCTCCAGCTCTCG-3′
*HOXB6* F	5′-TCCCCTCCCAATGAGTTCCT-3′
*HOXB6* R	5′-ACTCCTGCCCGCTGGC-3′
*PRDM16* F	5′-TGCCGCACGCAGATCA-3′
*PRDM16* R	5′-GGGAGGAGGTAGTGCTGAACAT-3′
*MECOM* F	5′-CGGAGTGTGGCAAAACGTT-3′
*MECOM* R	5′-GCTGTGGATGTGCTTGTGTTGT-3′
*NKX2-3* F	5′-GGTTCCAGAATCGCAGGTACAA-3′
*NKX2-3* R	5′-GCGCCAAGCTCCAGAGACT-3′
*VENTX* F	5′-GGCTGGCCAGGGAGATG-3′
*VENTX* R	5′-TGCGGCGATTCTGAAACC-3′
*UTF* F	5′-GACCAGCTGCTGACCTTGAA-3′
*UTF* R	5′-CTGCCCAGAATGAAGCCCA-3′
*ASL* F	5′-CAGCATGGATGCCACTAGTGA-3′
*ASL* R	5′-CACAGCGAAGCCCAGAACA-3′
*GAPDH* F	5′-AATCCCATCACCATCTTCCA-3′
*GAPDH* R	5′-TGGACTCCACGACGTACTCA-3′
**Fragment analysis primers**	
*FLT3* exon14 F	5′-FAM-AGCAATTTAGGTATGAAAGCCAGCTA-3′
*FLT3* exon14 R	5′-CTTTCAGCATTTTGACGGCAACC-3′

**Table 2:** Individual characteristics and clinical outcome of AML patients with *NUP98::NSD1* fusion transcript.

Patient No.	Age (in Years)	Sex	FAB	WBC Count at Diagnosis (WBC Count /L)	BM Blast (%)	FLT3-ITD Status	Treatment Protocol	Induction Chemo Response	HSCT	Outcome
**Adult #1**	57	M	M4	19.2 * 10^9^	70%	FLT3-ITD Positive (AR 0.35)	3 + 7 f/b HAM	Induction failure	No	Expired due to severe sepsis & respiratory failure
**Adult #2**	64	F	M2	12.8 * 10^9^	95%	FLT3-ITD Positive (AR 0.35)	Azacitidine + Venetoclax f/b HAM	Induction failure	No	Died due to disease progression
**Adult #3**	33	M	M3	20 * 10^9^	95%	FLT3-ITD Positive (AR 0.31)	3 + 7 + Midostaurin	Induction failure	No	Died due to intracranial bleed.
**Pediatric #1**	15	M	M2	19.9 * 10^9^	55%	FLT3-ITD Positive (AR 0.36)	3 + 7 + Midostaurin	Achieved remission	Yes	Disease relapsed 7 months after allogenic stem cell transplant
**Pediatric #2**	15	F	M2	18.6 * 10^9^	71%	FLT3-ITD Positive (AR 0.21)	3 + 7 + Midostaurin	Induction failure	No	Expired due to MDR sepsis (kliebsella pneumoniae)
**Pediatric #3**	12	F	M1	17.9 * 10^9^	90%	FLT3-ITD Positive (AR 0.37)	3 + 7 + Midostaurin f/b HAM+ Midostaurin	Induction failure	Yes	Disease relapsed 4 months after allogenic stem cell transplant

Abbreviations: FAB—French American British; WBC-white blood cell; 3 + 7—3 days daunorubicin + 7 days cytosine arabinoside; HAM—high dose cytosine arabinoside + mitoxantrone; FLAG—ida-fludarabine, cytosine arabinoside, idarubicin; HSCT—hematopoietic stem cell transplant; f/b—followed by; BM—bone marrow; MDR—multidrug resistance; AR—allelic ratio; FLT3-ITD—FLT3 internal tandem duplication.

## Data Availability

Not applicable.
